# A metalloprotease secreted by an environmentally acquired gut bacterium hinders *Borrelia afzelii* colonization in *Ixodes ricinus*

**DOI:** 10.3389/fcimb.2024.1476266

**Published:** 2024-10-10

**Authors:** Adnan Hodžić, Gorana Veinović, Amer Alić, David Seki, Martin Kunert, Georgi Nikolov, Ratko Sukara, Jovana Šupić, Snežana Tomanović, David Berry

**Affiliations:** ^1^ Centre for Microbiology and Environmental Systems Science, Department of Microbiology and Ecosystem Science, Division of Microbial Ecology, University of Vienna, Vienna, Austria; ^2^ Institute for Medical Research, National Institute of Republic of Serbia, University of Belgrade, Belgrade, Serbia; ^3^ Department of Clinical Sciences of Veterinary Medicine, Faculty of Veterinary Medicine, University of Sarajevo, Sarajevo, Bosnia and Herzegovina; ^4^ Joint Microbiome Facility of the Medical University of Vienna and the University of Vienna, Vienna, Austria

**Keywords:** *Borrelia afzelii*, enhancin, gut microbiome, *Ixodes ricinus*, peritrophic matrix

## Abstract

Although the importance of the microbiome in the context of tick biology and vector competence has recently come into a broader research focus, the field is still in its infancy and the complex ecological interactions between the tick residential bacteria and pathogens are obscure. Here, we show that an environmentally acquired gut bacterium has the potential to impair *Borrelia afzelii* colonization within the tick vector through a secreted metalloprotease. Oral introduction of either *Bacillus cereus* LTG-1 isolate or its purified enhancin (*Bc*Enhancin) protein significantly reduces *B. afzelii* burden in the guts of *Ixodes ricinus* ticks. This effect is attributed to the ability of *Bc*Enhancin to degrade a glycan-rich peritrophic matrix (PM), which is a gut protective barrier essential for *Borrelia* survival. Our study highlights the importance of the gut microbiome in determining tick vector competence and provides a deeper mechanistic insight into the complex network of interactions between *Borrelia*, the tick, and the tick microbiome.

## 1 Introduction

Lyme borreliosis (LB) is an emerging and the most prevalent vector-borne infectious disease in the temperate regions of the Northern Hemisphere. It is caused by the spirochetes of the *Borrelia burgdorferi* sensu lato complex, which includes at least 20 different species, six of which are recognized human pathogens (Steinbrink et al., 2022). These extracellular bacteria are engaged in a complex enzootic life cycle that involves vertebrate reservoir hosts, mainly rodents and birds, and ticks of the genus *Ixodes*. The castor bean tick, *Ixodes ricinus*, is the main vector of LB in Europe, where most human cases are caused by *Borrelia afzelii*, *Borrelia garinii*, and less often by *B. burgdorferi* sensu stricto (hereafter *B. burgdorferi*) (Steere et al., 2016).

Borrelial pathogens have evolved multiple strategies for survival in the tick vector and the vertebrate host. After ingestion from an infected vertebrate, the spirochetes colonize the tick gut and remain attached to the epithelium for several months until the next blood meal. During this phase, *Borrelia* must overcome several barriers to survive a hostile gut environment, including humoral and cellular immune responses, endocytic digestion, and toxic products associated with blood digestion (Estrada-Peña et al., 2018). To confront these challenges, *Borrelia* induces extensive transcriptional changes and modulates the gut environment (Kurokawa et al., 2020). Interactions between the pathogen and the tick gut are thus critical for colonization as well as spirochete transmission to a mammalian host (Kurokawa et al., 2020; Pal et al., 2021). Mounting evidence suggests that residential gut bacteria may also affect the colonization of pathogens within the tick vector, but the molecular mechanisms of these interactions are not fully elucidated (Narasimhan et al., 2014; Gall et al., 2016; Abraham et al., 2017; Narasimhan et al., 2017; Ross et al., 2018; Landesman et al., 2019; Sperling et al., 2020; Narasimhan et al., 2022; Wu-Chuang et al., 2023). This is of particular importance for *Borrelia* species considering that they lack interbacterial effector and immunity genes required for survival in a polymicrobial environment, which makes them highly susceptible to inhibition by cohabiting bacteria (Ross et al., 2018). In line with this observation, a higher abundance and diversity of the microbial communities in wild-caught ticks compared to laboratory-reared ticks have been associated with increased colonization resistance to *B. afzelii* (Jacquet et al., 2017; Guizzo et al., 2020). Moreover, the abundance of *Borrelia* negatively correlates with the increased burden of certain bacterial taxa, such as *Bacillus*, *Pseudomonas*, and Enterobacteriaceae (Ross et al., 2018; Landesman et al., 2019; Grandi et al., 2023; Wu-Chuang et al., 2023).

The tick gut microbiome is important for the formation of an acellular and glycan-rich structure known as a peritrophic matrix (PM) (Narasimhan et al., 2014). The arthropod PM is analogous to the vertebrate mucosal layer, and it serves as a physical barrier that separates the gut lumen from the epithelia and protects the epithelium from invading pathogens and their toxins (Kitsou et al., 2021). In *Ixodes* ticks, the PM is a transient structure that forms in the early stages of blood feeding, usually 9-12 hours after tick attachment, and which remains intact for several days (Zhu et al., 1991; Grigoreva and Amosova, 2004). Dysbiosis induced by environmental changes has been shown to interrupt PM formation by diminishing the expression of peritrophin, a core structural component of the PM, and the compromised integrity of the protective barrier in *Ixodes scapularis* ticks impairs the ability of *B. burgdorferi* to colonize the gut (Narasimhan et al., 2014). An intact PM, therefore, appears to be decisive for *Borrelia* persistence within the tick vector as it serves as a shield that protects the spirochetes from detrimental luminal components during colonization of the gut epithelium (Kariu et al., 2013; Narasimhan et al., 2014; Kurokawa et al., 2020; Kitsou et al., 2021; Yang et al., 2021).

These findings prompted us to investigate whether PM-degrading proteins from tick-associated bacteria can affect *B. afzelii* colonization. Our results show that enhancin, a metalloprotease with mucinase activity that is produced and secreted by a *Bacillus cereus* strain isolated from *I. ricinus* ticks (*Bc*Enhancin), reduces *B. afzelii* levels by impairing the integrity of the PM.

## 2 Materials and methods

### 2.1 Ticks

Unfed and pathogen-free *I. ricinus* females were obtained from a tick colony at the Insect Services GmbH, Berlin, Germany. Upon arrival, the ticks were kept in a desiccator at 22°C and ~ 97% relative humidity under a 14:10-hour light-dark photoperiod for at least seven days before feeding experiments. A capillary feeding technique was employed for tick infections, so animal experimentations and ethical permissions were not required.

### 2.2 Isolation and characterization of tick gut bacteria

Host-seeking *I. ricinus* ticks were collected from three different locations in the city of Vienna by flagging the vegetation (**Supplementary Figure S1**). Overall, 524 ticks (415 nymphs, 67 females, and 42 males) were collected between April and July 2023 and processed for bacteria isolation. After identification, ticks were surface sterilized with 1% sodium hypochlorite solution for 3 min, 70% ethanol for 1 min, and then rinsed in three successive baths of sterile water (Guizzo et al., 2022). The last rinse water was plated and incubated under the same conditions as gut samples to control the efficacy of the decontamination protocol. The guts were aseptically dissected under a stereomicroscope (Olympus SZ61, Japan) using sterile surgical blades (No. #11, Integra Miltex, Japan) and fine tip forceps and then homogenized either individually or in pools (three adults or five nymphs) in 200 μl sterile phosphate-buffered saline (PBS). The gut homogenates were streaked onto Brain heart infusion (BHI) agar (Oxoid, UK) supplemented with 5 g/l yeast extract (Oxoid, UK) and 5 mg/l hemin (Sigma-Aldrich, MO, USA), and incubated at 22°C and 37°C under aerobic and anaerobic (anaerobic tent, 85% N_2_, 10% CO_2_, 5% H_2_) conditions for 72 h. Two plates per sample were used for each incubation condition. Individual bacterial colonies with distinct morphologies were picked from the plate, dissolved in nuclease-free water, and used as a template for amplification of the 16S rRNA gene by colony PCR (Riva et al., 2023) using the universal eubacterial primers 27f and 1492r (**Supplementary Table S1**). Amplification was conducted in a T100 Thermal Cycler (Bio-Rad Laboratories, Germany) under the following conditions: initial denaturation 95°C for 4 min followed by 35 cycles of 95°C for 30 s, 51°C for 30 s, 72°C for 1.5 min, and a final elongation at 72°C for 10 min. PCR products were separated by electrophoresis on a 1.5% agarose gel stained with GelRed (Biotium, CA, USA). Purification of the amplicons and Sanger sequencing were conducted by Microsynth AG (Vienna, Austria). The DNA sequences were edited with BioEdit software v.7.2.5 (Hall, 1999) and EzBioCloud’s identification service (ezbiocloud.net) was employed for similarity-based searches against quality-controlled databases of 16S rRNA gene sequences (Yoon et al., 2017). The growth condition and the isolation source for each bacterial isolate are summarized in **Supplementary Table S2.**

### 2.3 Identification of bacterial enhancins

The Pfam 36.0 database (Mistry et al., 2021) was searched to ascertain the occurrence and the taxonomic distribution of enhancin proteins, which are metalloproteases known to promote bacterial and viral infections by degrading the polysaccharide layer of the invertebrate PM (Wang and Granados, 1997; Peng et al., 1999; Galloway et al., 2005; Fang et al., 2009; Garcia-Gonzalez and Genersch, 2013; Wu et al., 2019; Nakamura et al., 2021). Identification and characterization of enhancins across Bacteria superkingdom were performed by searching the Peptidase M60, enhancin and enhancin-like (M60-like; PF13402), and Putative mucin or carbohydrate-binding module (Mucin_bdg; PF03272) domains. These two domains are the defining feature of the mucin-degrading enhancins (Wang and Granados, 1997; Nakjang et al., 2012). Candidate proteins were checked individually to reduce the number of false positive entries. The protein structure was assessed using the SMART research tool (Letunic et al., 2021) in Normal Mode, which contains Swiss-Prot, SP-TrEMBL, and stable Ensembl proteomes. Signal peptides were additionally predicted by SignalP-5.0 (cbs.dtu.dk/services/SignalP). Schematic representation of the enhancin proteins was done by entering “M60-like” AND Pfam: Mucin_bdg” in the domain selection and the domain architecture query was restricted to Bacteria. Finally, a Newick tree was generated based on National Center for Biotechnology Information (NCBI) taxonomy (ncbi.nlm.nih.gov) and displayed in iTOL v.6 (Letunic and Bork, 2021).

Nucleotide sequences of the enhancin encoding genes were retrieved from the genomes of the selected bacterial representatives available in the GenBank^®^ database. To detect the *enhancin* gene in the *B. cereus* LTG-1 strain, a PCR assay targeting a 952 bp long fragment was developed. The PCR mixture contained 12.5 μl of 2X DreamTaq PCR Master Mix (Thermo Fischer Scientific, IL, USA), 1 μl of each BcEnhc-F and BcEnhc-R primer (**Supplementary Table S1**), 5 μl of DNA template, and PCR grade water up to 25 μl. The amplification program consisted of initial denaturation at 95°C for 3 min, followed by 35 cycles of denaturation at 95°C for 30 s, 60°C for 30 s, 72°C for 1 min, and a final elongation at 72°C for 7 min. The resulting PCR product was evaluated by gel electrophoresis and submitted for bidirectional DNA sequencing using the amplification primers (Microsynth AG, Austria).

### 2.4 Phylogenetic tree reconstruction

Bacterial 16S rRNA gene sequences obtained from bacterial isolates were aligned in BioEdit software v.7.2.5 using ClustalW with the default settings (Hall, 1999). Poorly aligned regions were edited manually, and the phylogeny was calculated by the Maximum Likelihood (ML) method in the bioinformatics software MEGA v.7.0 (Kumar et al., 2016). Best-fit nucleotide substitution model (T92+G+I) was selected according to AICc values (Akaike information criterion corrected), and the tree topology was completed using the Nearest-Neighbor-Interchange (NNI) heuristic model. Internal nodes of the tree were estimated with 1,000 bootstrap replicates. An ML tree of the protein alignment was computed based on the WGM model (Whelan and Goldman, 2001) implemented in MEGA v.7.0 (Kumar et al., 2016). The dataset was assessed with 100 bootstrap replicates.

### 2.5 Production of recombinant *Bc*Enhancin

A recombinant form of the *B. cereus* enhancin (r*Bc*E) was synthesized by GenScript Biotech, Netherlands. Briefly, the coding sequence of the WBV46172.1 protein without its putative signal peptide was cloned into the pET30a template vector with *Nde*I and *Hin*dIII restriction sites and expressed in the *E. coli* BL21 (DE3) system. The soluble protein with a C-terminal polyhistidine tag was induced at two different expression conditions, 37°C for 4 h and 15°C for 16 h, and then purified in a single step by Ni resin. The expression level of the recombinant protein and purity were analyzed under reducing conditions by SDS-PAGE and Western Blot. The purified r*Bc*E was aliquoted and stored at - 80°C until used for tick feeding.

### 2.6 Tick capillary feeding and bacterial infection

**Figure 1:**
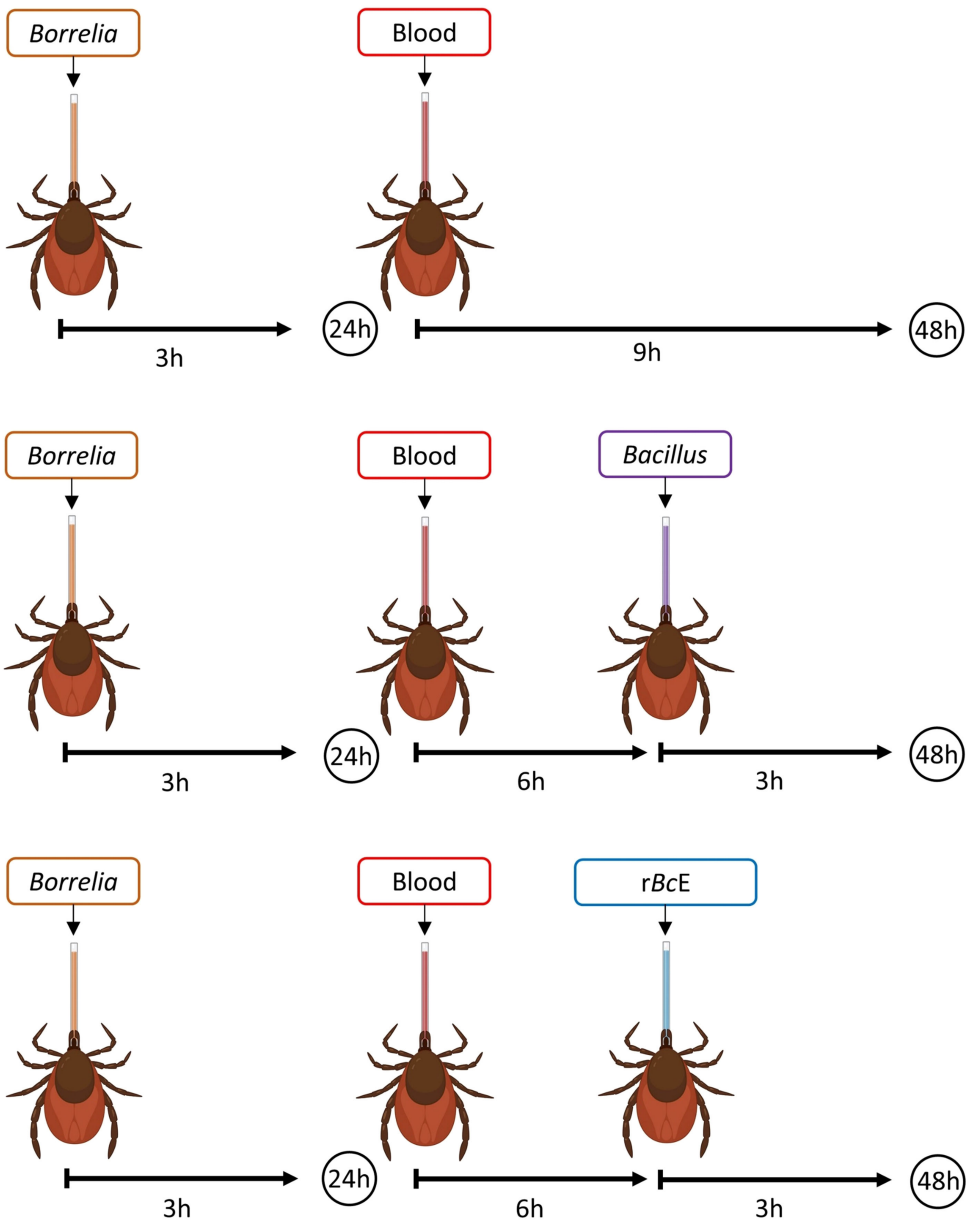
A schematic illustration of the experimental design. Ticks were infected with *B. afzelii* RS 163_11i using glass capillaries and after the 24-hour recovery period, they were fed either with *B. cereus* LTG-1 or *B. licheniformis* SP-3 bacteria suspensions or the purified *B. ereus* enhancin (r*Bc*E) protein. Bacterial levels were assessed 48 hours after feeding by qPCR. The figure was created with BioRender.com.

Depending on the experimental setup, female *I. ricinus* ticks were fed by blood, bacteria suspension, or purified protein using a modified capillary feeding technique (**Figure 1**) (Kim et al., 2021). Ticks from all groups were first infected with the wild-type *B. afzelii* strain RS 163_11i initially isolated from an *I. ricinus* tick in Serbia (Ćakić et al., 2019). An aliquot of frozen spirochetes was thawed at room temperature, transferred into sterile glass tubes, and incubated in 6 ml of Barbour-Stoenner-Kelly (BSK-H) medium supplemented with 6% rabbit serum (Sigma-Aldrich, St. Louis, MO, USA) at 33°C. *Borrelia* culture was grown until the cells reached a concentration of ~ 1 x 10^7^/ml. The spirochete number was estimated by dark field microscopy using a Neubauer counting chamber (Wertheim, Germany) as previously described (Veinović et al., 2016). Sterile 5 μl glass capillaries (Drummond, PA, USA) filled with the culture were placed under a stereomicroscope over the hypostomes of ticks immobilized on a double-sided adhesive tape mounted on a microscopic slide (**Supplementary Figure S2**). Ticks were carefully removed from the tape after 3 h of feeding, placed in sterile 5 ml tubes with holes, and kept in a desiccator at 22°C and ~ 97% relative humidity for 24 h. After the 24-h recovery period, ticks were fed either by fresh defibrinated sheep blood (Oxoid, UK) alone or the blood in combination with bacteria suspension or the purified r*Bc*E protein depending on the group (**Figure 1**). *Bacillus cereus* LTG-1 and *B. licheniformis* SP-3 isolates were used for tick oral infection. The glycerol stocks of the bacteria were inoculated into Luria-Bertani (LB) broth (Carl Roth, Germany) and incubated overnight at 37°C with shaking. The overnight cultures were diluted in LB broth to obtain a standardized OD_600_ of 0.02 and incubated for an extra 2 h. The bacteria suspensions were mixed with the sheep blood (50:50 v/v) and used in two separate feeding experiments. Ticks from the r*Bc*E group received a mixture of the blood and the purified r*Bc*E at the final concentration of 15 μg/ml. This concentration is comparable to the amount of the recombinant enhancin used for mosquito feeding experiments conducted by Wu and colleagues (2019). Control ticks (mock) were fed with r*Bc*E inactivated at 70°C for 30 min. Tick feeding was performed in a humidified chamber in an incubator at 32°C. After feeding, ticks were separated by groups in sterile 5 ml tubes and maintained in the desiccator for 48 h before being processed for analysis.

### 2.7 Quantification of bacterial loads by qPCR

Prior to dissection, ticks were washed in 70% ethanol and sterile MQ water as described above. The wash-off water was used for DNA extraction and served as a control in downstream analyses. The individual tick guts were digested with proteinase K at 56°C overnight and then subjected to genomic DNA extraction using a QIAamp DNA Mini kit (Qiagen, Germany). The spirochete load was assessed by a probe-based qPCR that targets a 132 bp fragment of the *fla* gene that encodes *Borrelia* flagellin (Schwaiger et al., 2001). An additional qPCR targeting the mitochondrial 16S rRNA gene of *Ixodes* was run for DNA extraction control and data normalization purposes (Becker et al., 2023). Both qPCRs were conducted in 20 µl reactions containing GoTaq Probe qPCR Mastermix (Promega, WI, USA), 10 pmol of each primer, 5 pmol of Taqman probe, 5 μl DNA template, and nuclease-free water. Primer and probe sequences are listed in **Supplementary Table 1**. All qPCRs were performed in a CFX96 Real-Time PCR cycler (Bio-Rad Laboratories, Germany). The cycling conditions were identical for both PCR assays and consisted of 2 min at 95°C for denaturation, followed by 50 cycles of 15 sec at 60°C and 1 min at 95°C. Two technical replicates were tested, and no template controls were included in each PCR run. Relative abundance of spirochetes was normalized to the tick 16S rRNA reference gene and calculated using the 2^-ΔΔCt^ method (Pfaffl, 2001).

Quantification of total bacteria load in individual tick guts was assessed by a SYBR Green qPCR using the universal 341F/785R primers that target the hypervariable V3-V4 region of the 16S rRNA gene (Klindworth et al., 2013). The data were normalized to the tick 16S rRNA gene using the Ixo16-F/Ixo16S-R primers (Pfaffl, 2001; Becker et al., 2023). The PCRs were performed with the iQ SYBR Green Supermix (Bio-Rad Laboratories, Germany) following the manufacturer’s recommendations.

### 2.8 Bactericidal assay

To assess the potential borreliacidal effect of the purified r*Bc*E, we performed an antimicrobial susceptibility *in vitro* test as previously described (Veinović et al., 2021). Briefly, *B. afzelii* RS 163_11i strain was grown to log phase and the bacterial suspensions (4 x 10^5^ spirochetes/ml) were incubated at 33°C either with active or heat-inactivated r*Bc*E protein at three different concentrations (15 µg/ml, 30 µg/ml, and 60 µg/ml) or the ceftriaxone antibiotic (4 µg/ml), which was used as a positive control. Untreated bacterial culture served as a negative control. Proteins and the antibiotic preparations were filtered through 0.22 µm syringe filters (Sartorius Stedim Biotech GmbH, Germany) before incubation. The number, viability (%), and morphology of spirochetes were assessed by dark field microscopy after 24 and 48 h of incubation.

### 2.9 Whole-mount fluorescence *in situ* hybridization

A modified FISH protocol (Duron et al., 2018) was employed for the visualization of bacteria in tick guts. Whole ticks were fixed in a 4% paraformaldehyde solution 48 h post-feeding. To enable faster penetration of the fixative, the tick cuticula was pierced in several places with a sterile needle. After 3 days of fixation at 4°C, ticks were dissected under a stereomicroscope. The extracted guts were incubated overnight at 46°C in 200 μl of hybridization buffer (36 µl of 5M NaCl, 4 µl of 1M Tris-HCl, 0.02 g of dextran sulfate, 20 µl of blocking agent, 0.2 µl of 10% SDS, 40 µl of formamide, and 100 µl of MQ water) containing the Borr4 (Hammer et al., 2001) oligonucleotide probe labeled with Cy3 dyes at both ends (**Supplementary Table S1**). Next, the samples were washed with the washing buffer (43 µl of 5M NaCl, 20 µl of 1M Tris-HCl, 10 µl of 0.5M EDTA, 1 µl of 10% SDS, and 926 µl of MQ water) at 48°C for 30 min and additionally with 1 x PBS for 15 min at room temperature to remove the excess probes from the tissue. Counterstaining with DAPI (10 μg/ml in PBS) was performed in the dark for 8 min. Finally, the guts were washed with ice-cold MQ water and placed on a microscope slide, mounted with an antifade mounting media (Citifluor, PA, USA), and covered with a cover slip. The slides were examined with the Leica DMi8 Thunder Epifluorescence microscope (Leica Microsystems, Germany) using 20X dry, 63X glycerol, and 100X oil objective lens. The Leica LAS X microscope software (Leica Thunder Imager, Leica Microsystems, Germany) scanning the DAPI and Cy3 fluorescence channels with the Maximum Intensity Projection of 50 Z-stack images was used for FISH image acquisition.

### 2.10 Tick sectioning and Periodic acid Schiff’s staining

Tick histology and PAS staining were performed at the Department of Clinical Sciences of Veterinary Medicine in Sarajevo following the protocol published by Narasimhan and colleagues (2014). Briefly, formalin-fixed ticks were routinely processed, embedded in paraffin, and cut at 3-6 µm sections. The sections were deparaffinized, stained with PAS (Sigma-Aldrich, MO, USA), and visualized by light microscopy (Olympus BX51, Japan) at 40X magnification. Images were acquired using computer image analysis software (Cell Digital Imaging Software, Olympus, Japan). At least 6-10 sections per tick were examined.

### 2.11 Gene expression by RT-qPCR

RNA was extracted from pooled tick guts (five guts per pool) using a Total RNA Purification kit (Norgen Biotek, Canada) following the manufacturer’s instructions. Prior to extraction, the guts were homogenized in 600 μl of Buffer RL supplemented with β-mercaptoethanol (Sigma-Aldrich, Austria) by passage through 24- and 27-gauge needles (Knorr et al., 2021). The RNA extraction from *B. cereus* LTG-1 culture was done following the protocol for bacterial cells with an additional lysozyme step (Sigma-Aldrich, Austria). Quantification of RNA was accomplished with the Qubit 4 Fluorometer (Thermo Fischer Scientific, IL, USA) and Qubit RNA Broad Range Assay kit (Thermo Fischer Scientific, IL, USA). The isolates were additionally purified and concentrated with the RNA Clean & Concentrator kit (Zymo Research, CA, USA). Subsequently, the RNA samples free of contaminating DNA (Turbo DNA-free kit, Ambion, USA) were reverse transcribed to cDNA with the High-Capacity cDNA Reverse Transcription kit (Applied Biosystems, CA, USA) and stored at - 20°C until use. To examine whether r*Bc*E affects tick gut immune response, the relative transcript levels of the major immune genes and genes related to the PM formation were determined by RT-qPCR. Primer sequences are listed in **Supplementary Table S1**. The amplification was carried out in a CFX96 Real-Time PCR cycler (Bio-Rad Laboratories, Germany) with an initial denaturation at 95°C for 5 min followed by 35 cycles at 95°C for 15 sec and 63°C for 30 sec. A melt curve analysis was performed to check the specificity of the resulting amplicons, and a no-template control was included for each gene. The PCR mixture was prepared in a final volume of 20 μl using iQ SYBR Green Supermix (Bio-Rad Laboratories, Germany), 0.8 μl of each primer, 1 μl of cDNA, and nuclease-free water. The relative gene expressions were calculated using the 2^-ΔΔCt^ method (Pfaffl, 2001) and normalized to the levels of the *I. ricinus* elongation factor 1-alpha (*elf-1a*) housekeeping gene.

### 2.12 Microbiota analysis by 16S rRNA gene-targeted amplicon sequencing

Amplicon sequencing was performed at the Joint Microbiome Facility of the University of Vienna and the Medical University of Vienna (project ID: JMF-2311-10). Briefly, the hypervariable V3-V4 regions of the bacterial 16S rRNA genes were amplified with the primers 341F and 785R containing 16 bp head adapters (H1: 5′-GCTATGCGCGAGCTGC-3′, H2: 5′-TAGCGCACACCTGGTA-3′) in the first PCR step, followed by a second barcoding PCR step performed with 12 bp unique dual barcodes (Herbold et al., 2015; Pjevac et al., 2021). After each amplification, the products were purified and normalized with the SequalPrep^™^ Normalization Plate kit (Invitrogen), and the second-step amplicons were pooled and concentrated on columns with the innuPREP PCRpure Kit (Analytik Jena, Germany). Sequence library preparation was done using the Illumina TruSeq DNA Nano Kit (Illumina, CA, USA) and libraries were sequenced in paired-end mode (2 × 300 nt; v3 chemistry) on an Illumina MiSeq (Illumina, CA, USA). Next, amplicon pools were extracted from the raw sequencing data applying default parameters in FASTQ workflow in BaseSpace (Illumina), PhiX sequences were decontaminated using BBDuk (BBtools) (Bushnell et al., 2017), and the sequences were demultiplexed with the Python package demultiplex by permitting one mismatch for barcodes and two mismatches for linkers and primers (Herbold et al., 2015; Pjevac et al., 2021). FASTQ reads 1 and 2 were trimmed at 230 bp with allowed expected errors of 4 and 6, respectively, paired-end reads were merged and ASVs were subsequently inferred with the Divisive Amplicon Denoising Algorithm (DADA2) (Callahan et al., 2016a, Callahan et al., 2016b). Taxonomy was assigned with the SINA v.1.6.1 classifier (Pruesse et al., 2012) against the SILVA SSU database SSU Ref NR 99 v.138.1 (doi.org/10.5281/zenodo.3986799). Taxa represented at less than 1% relative abundance were excluded from the analyses to rule out potential environmental contaminants (Narasimhan et al., 2022).

### 2.13 Statistical analysis

Statistical analyses were performed using GraphPad Prism10 (GraphPad Software Inc., CA, USA) and R v.4.0 statistical software. The differences between the control and experimental groups were analyzed using a nonparametric two-tailed Mann-Whitney *U* test. 16S rRNA data analyses were performed using R packages rstatix v.0.7.0, ampvis v.2.0 (Allaire, 2012), and ggplot2 v.3.3.3 (Wickham, 2009). The Shapiro-Wilk test was performed to test for normality of the data, and the result of this determined whether *t* tests or Wilcoxon tests were used. All *p* values were adjusted via fdr correction. Alpha and beta diversity indices were calculated using the R package vegan v.2.5 (Oksanen et al., 2013). Differences were considered significant if *p* < 0.05.

## 3 Results

### 3.1 Bacteria residing in the guts of *I. ricinus* ticks express enhancins

**Figure 2:**
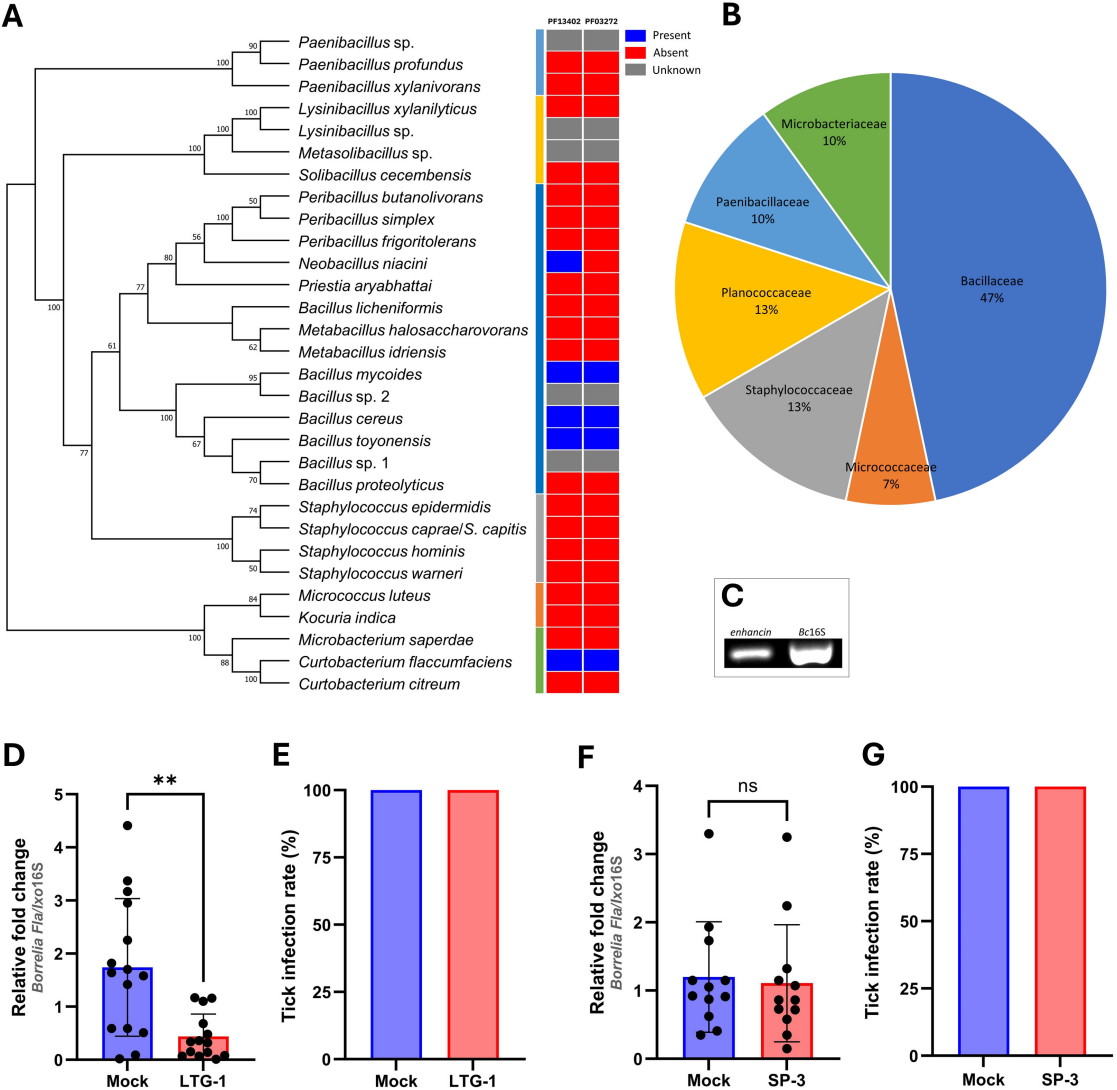
An enhancin-containing bacterium affects *Borrelia* colonization in the tick gut. **(A)** Bacteria identified by culture-dependent approach in individual or pooled guts of nymphal, male, and female ticks. An ML bootstrap tree of the 16S rRNA nucleotide sequences of bacterial isolates (1329 bp). The tree with the highest log likelihood (13903.286) is shown. Bootstrap values based on 1,000 replicates are indicated at the nodes (only values higher than 50% are shown). Color strip: Presence and absence of the Peptidase M60, enhancin and enhancin-like (PF13402) and Putative mucin or carbohydrate-binding module (PF03272) conserved domains across the phylogeny of 30 bacterial isolates based on the Pfam domain analysis. **(B)** Prevalence of the bacteria (%) isolated from *I*. *ricinus* ticks at the family level. **(C)** Expression of *enhancin* in bacteria suspension (*B. cereus* LTG-1) assessed by RT-PCR. **(D, E)** *B*. *afzelii* load and the infection rate (%) in guts of female *I*. *ricinus* ticks co-infected with *B. cereus* LTG-1 strain. One dead tick was excluded from the analysis. **(F, G)** *B*. *afzelii* load and the infection rate (%) in guts of female *I*. *ricinus* ticks co-infected with *B*. *licheniformis* SP-3. **(D, F)** Each data point represents an individual gut. Results are presented as mean ± SD of two technical replicates. Normalized Ct values were compared between groups by a nonparametric two-tailed Mann-Whitney *U* test (ns, not significant, ^**^ *p* ≤ 0.01).

To understand the molecular mechanisms by which tick gut bacteria can antagonize the growth of *Borrelia* species, we first isolated cultivable bacteria from questing *I. ricinus* nymphs, males, and females collected from three locations in Vienna, Austria (**Supplementary Figure S1**). Overall, 30 bacterial species representing 15 genera were isolated from individual (15.8%) or pooled tick guts (39.6%) and almost half of the isolates belonged to the family Bacillaceae (**Figures 2A, B**). All isolates were representatives of aerobic and/or facultative anaerobic Gram-positive bacteria commonly found on the skin and in the environment (**Supplementary Table S2**).

Representatives of five bacterial species identified in the present study for which complete annotated genomes are publicly available, namely *B. cereus*, *Bacillus mycoides*, *Bacillus toyonensis, Neobacillus niacini*, and *Curtobacterium flaccumfaciens* were found to contain putative enhancins by Pfam domain and genome analysis (**Figure 2A**; **Supplementary Figure S3**). Considering the ability of *B. cereus* enhancins to digest mucins of different arthropods (Fang et al., 2009; Wu et al., 2019), we selected *B. cereus* LTG-1 strain as a model organism for the following tick feeding experiments. Furthermore, *B. cereus* strains are commonly found in *Ixodes* ticks (Martin and Schmidtmann, 1998; Murrell et al., 2003; Kmet and Čaplová, 2019; Rousseau et al., 2021; Yang et al., 2021; Tóth et al., 2023). The capacity of *B. cereus* to produce enhancin appears to be strain-dependent as not all sequenced strains encode the *enhancin* gene (Nakjang et al., 2012). Hence, a PCR assay targeting a 952 bp fragment was used for the gene detection and characterization in our bacterial isolate. Sequence analysis and Basic Local Alignment Search Tool (blast.ncbi.nlm.nih.gov) revealed a 100% homology of the obtained nucleotide sequence with the M60 family metallopeptidase from the *B. cereus* PL22-16A soil isolate (GenBank^®^ accession number: CP115856.1). Together, these results indicate that bacteria inhabiting *I. ricinus* guts contain putative enhancins and suggest their potential to alter the structural integrity of the tick PM by digesting the mucous-like matrix.

### 3.2 Coinfection with *B. cereus* LTG-1 reduces *B. afzelii* load in the tick gut

To investigate whether enhancin-containing bacteria can influence *B. afzelii* persistence in the tick vector, infected *I. ricinus* females were fed with *B. cereus* LTG-1 via capillary feeding (**Figure 1**). Bacteria loads in individual tick guts were assessed 48 h post-feeding by qPCR. Oral introduction of the bacteria suspension, in which the *enhancin* gene was expressed (**Figure 2C**), reduced *B. afzelii* levels in comparison with the control ticks (Mann Whitney *U* test, *p* = 0.0015, **Figure 2D**). However, feeding of *B. cereus* LTG-1 was unable to completely clear *B. afzelii* infection (**Figure 2E**). One tick from the LTG-1 group died after feeding and it was excluded from further analyses. While twelve out of 14 guts from the bacteria-fed ticks were positive by qPCR, none of the control ticks contained detectable DNA of *B. cereus*. This result suggests that a transient bacterial association is sufficient to reduce *B. afzelii* load. The inability of *B. cereus* LTG-1 to stably colonize *I. ricinus* guts may be due to colonization resistance conferred either by the resident tick microbiota or by host immune defenses. To exclude the possibility that the reduction in *B. afzelii* was due to modulation of the gut microbiome or other factors unrelated to enhancin production, we fed ticks with another tick-associated *Bacillus* species, *Bacillus licheniformis*, which does not encode enhancin (**Figure 2A**). The spirochete loads and *Borrelia* infection rates were comparable between control ticks and those fed with *B. licheniformis* SP-3 suspension (Mann Whitney *U* test, *p* = 0.5223, **Figures 2F, G**). Taken together, our results suggest that gut colonization with *B. cereus* LTG-1 inhibits *B. afzelii* growth within the tick vector due at least in part to its secreted *Bc*Enhancin.

### 3.3 *Bc*Enhancin hinders *B. afzelii* colonization by compromising the structural integrity of the PM

**Figure 3:**
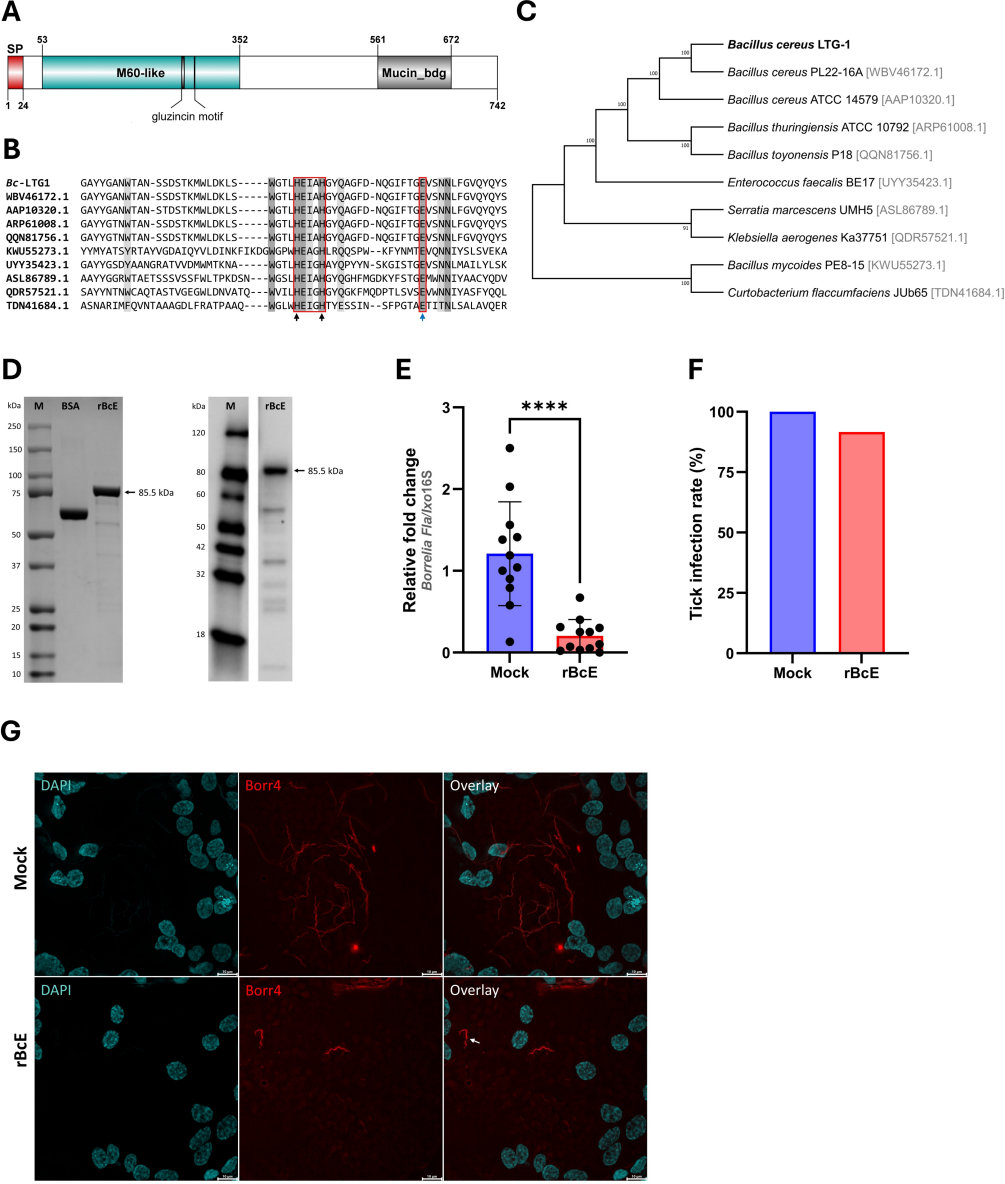
*Bc*Enhancin reduces *B*. *afzelii* growth by compromising the PM structural integrity. **(A)** Domain architecture of the putative *Bc*Enhancin protein. *Bc*Enhancin consists of the N-terminal signal peptide (SP) and two conserved domains. The M60-like domain contains a gluzincin motif (HEXXHX(8,24)E). The protein structure is visualized using DOG 2.0 Illustrator (Ren et al., 2009). **(B)** Partial amino acid sequence alignment of the M60-like domain identified in *I*. *ricinus*-related bacteria. Identical residues are shaded in dark grey, whereas similar residues are in light grey. The gluzincin with the typical zinc metallopeptidase motif (HEXXH) and the additional glutamate (E) residue is boxed. The arrows indicate the Zn^+^ binding (black) and catalytic (blue) residues. **(C)** An ML tree of the bacterial enhancin proteins based on the WGM model. GenBank^®^ accession numbers for each amino acid sequence are given in the brackets. Numbers at the nodes indicate bootstrap values based on 100 replicates (only values >50% are included). **(D)** Detection of the purified r*Bc*E by SDS-PAGE (left) and Western blot (right) under reducing conditions. Bovine serum albumin (BSA) served as a control in SDS-PAGE. r*Bc*E expression was detected by Western blot using a mouse anti-His monoclonal antibody. **(E, F)** Load of *B*. *afzelii* and the infection rate (%) in ticks fed by the heat-inactivated (mock) and active form of the r*Bc*E protein. Each data point represents an individual gut. Results are presented as mean ± SD of two technical replicates. Statistical significance was determined by a nonparametric two-tailed Mann-Whitney *U* test (^****^ *p* ≤ 0.0001). **(G)** Visualization of the spirochete burden and morphology in guts of female ticks by whole-mount FISH. Images represent five biological replicates per group. The arrow indicates a spirochete with altered morphology. *Scale-bar*: 10 μm.

**Figure 4:**
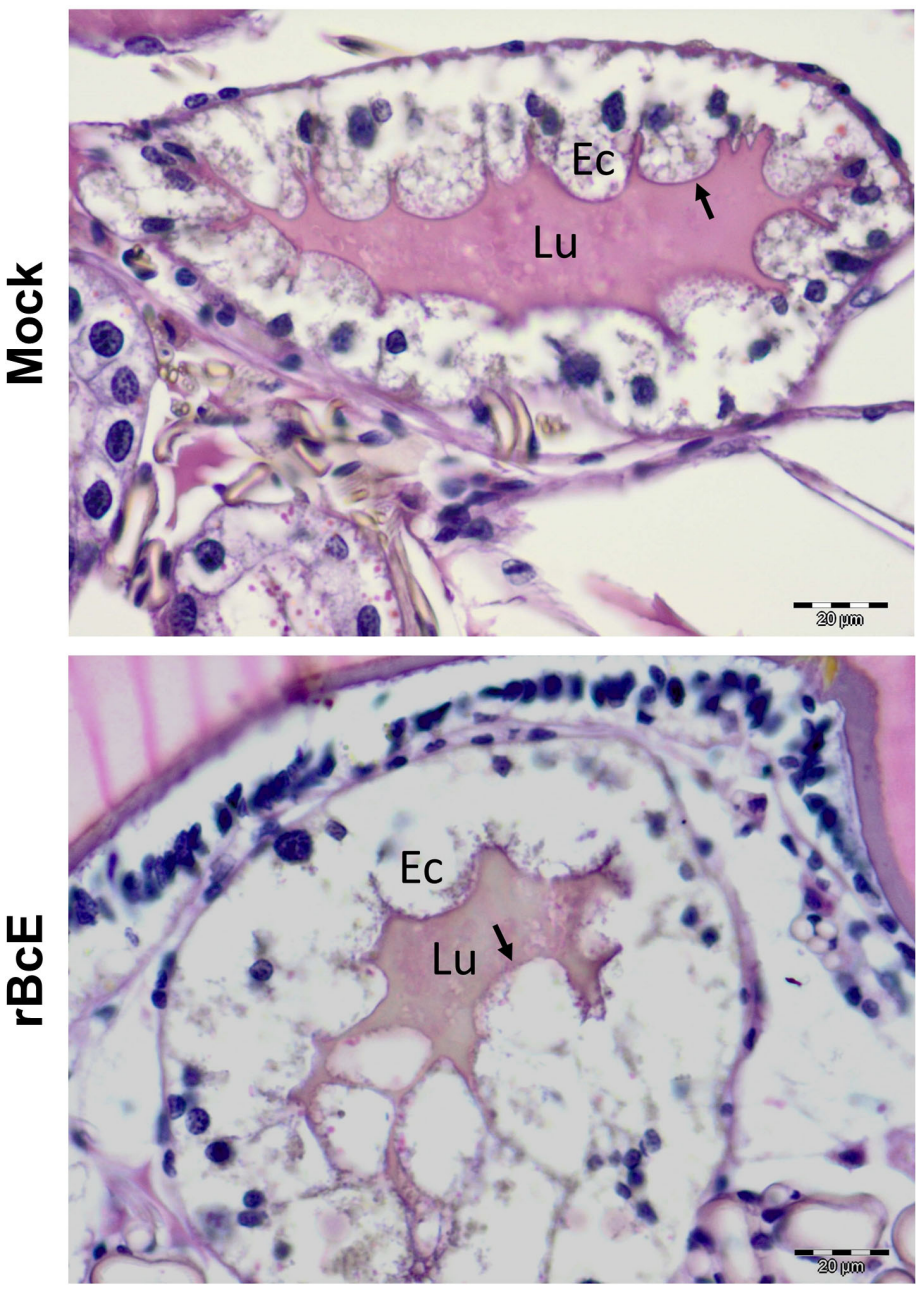
PAS staining of the tick gut sections showing the difference in the PM structure between control (mock) and r*Bc*E ticks. Results are shown for one representative of three biological replicates. Arrows indicate the PAS-positive PM layer. Lu, gut lumen; Ec, gut epithelial cell. *Scale-bar*: 20 μm.

The *Bc*Enhancin protein has a predicted molecular weight of 85.5 kDa and consists of an N-terminal signal peptide and M60-like (E-value: 5.55e-42) and Mucin_bdg (7.9e-36) domains (**Figure 3A**). The M60-like domain has a typical zinc metallopeptidase motif with an additional catalytic glutamic acid residue (HEXXHX(8,28)E) and it represents a mucin-active part of the protein (**Figures 3A, B**), whereas Mucin_bdg is the binding domain for enhancins and other similar metalloproteases (Wang and Granados, 1997; Nakjang et al., 2012). *Bc*Enhancin homologs are also found in other bacteria associated with *I. ricinus* ticks (Stojek and Dutkiewicz, 2004; Tveten et al., 2013; Lejal et al., 2021; Rousseau et al., 2021; Guizzo et al., 2022; Tóth et al., 2023) bearing 29.4% to 98.5% similarity at the protein level (**Figures 3B, C**). To experimentally test the hypothesis that secreted *Bc*Enahncin has the potential to impair *B. afzelii* colonization, we generated a recombinant version of the protein (r*Bc*E) using an *E. coli* BL21 (DE3) expression system and assessed the ability of the purified form to digest the tick PM *in vivo*. Sodium dodecyl-sulfate polyacrylamide gel electrophoresis (SDS-PAGE) and Western blot analyses revealed a single protein of the expected molecular weight with ≥ 90% purity (**Figure 3D**). The purified r*Bc*E was used for tick feeding (**Figure 1**) and the spirochete burden was determined by qPCR and FISH. The control group received an equal amount of heat-inactivated r*Bc*E. Ticks fed the purified protein showed significantly decreased *B. afzelii* levels (Mann Whitney *U* test, *p* < 0.0001, **Figure 3E**) and slightly lower infection rate (*p* > 0.05, **Figure 3F**) when compared to control ticks. All ticks survived the treatment and qualitative assessment of tick activity after feeding (i.e., mobility and questing activity) did not reveal any apparent deviation from normal tick behavior, suggesting that r*Bc*E does not have observable acute effects on tick fitness. The qPCR results were further confirmed by visualization of the spirochetes in the guts by FISH using a *Borrelia*-specific probe (Hammer et al., 2001). Clusters of spirochetes in the guts of control ticks could be seen in almost every microscopic field of view, whereas guts from r*Bc*E-fed ticks contained only a few scattered bacteria (**Figure 3G**). Besides the differences in the spirochete number and the spatial organization between the groups, a deviation from the typical flat-wave spirochete morphology was observed in the r*Bc*E group (**Figure 3G**), which might be indicative of impaired bacteria mobility (DeHart et al., 2021). *In vitro* assessment of the r*Bc*E on pathogen burden, viability, or morphology revealed that the suppressive effect of r*Bc*E is not due to direct bactericidal action (**Supplementary Figure S4**). To further examine the potential effect of r*Bc*E on the structural integrity of the PM, tick histology sections were stained with the PAS base stain, which specifically detects the glycan-rich layer of the PM in blood-feeding arthropods (Narasimhan et al., 2014; Abraham et al., 2017; Narasimhan et al., 2017; Wu et al., 2019). Ticks fed with r*Bc*E displayed thinner and fragmented PM 24 hours after oral administration in comparison to those treated with inactivated enzyme (**Figure 4**). Together, this suggests the ability of *Bc*Enhancin to affect *B. afzelii* colonization by impairing the structural organization of the PM and further reinforce the importance of this physical gut barrier for persistence of *Borrelia* within its arthropod vector.

### 3.4 *Bc*Enhancin treatment induces transcriptional changes in host immune response genes

**Figure 5:**
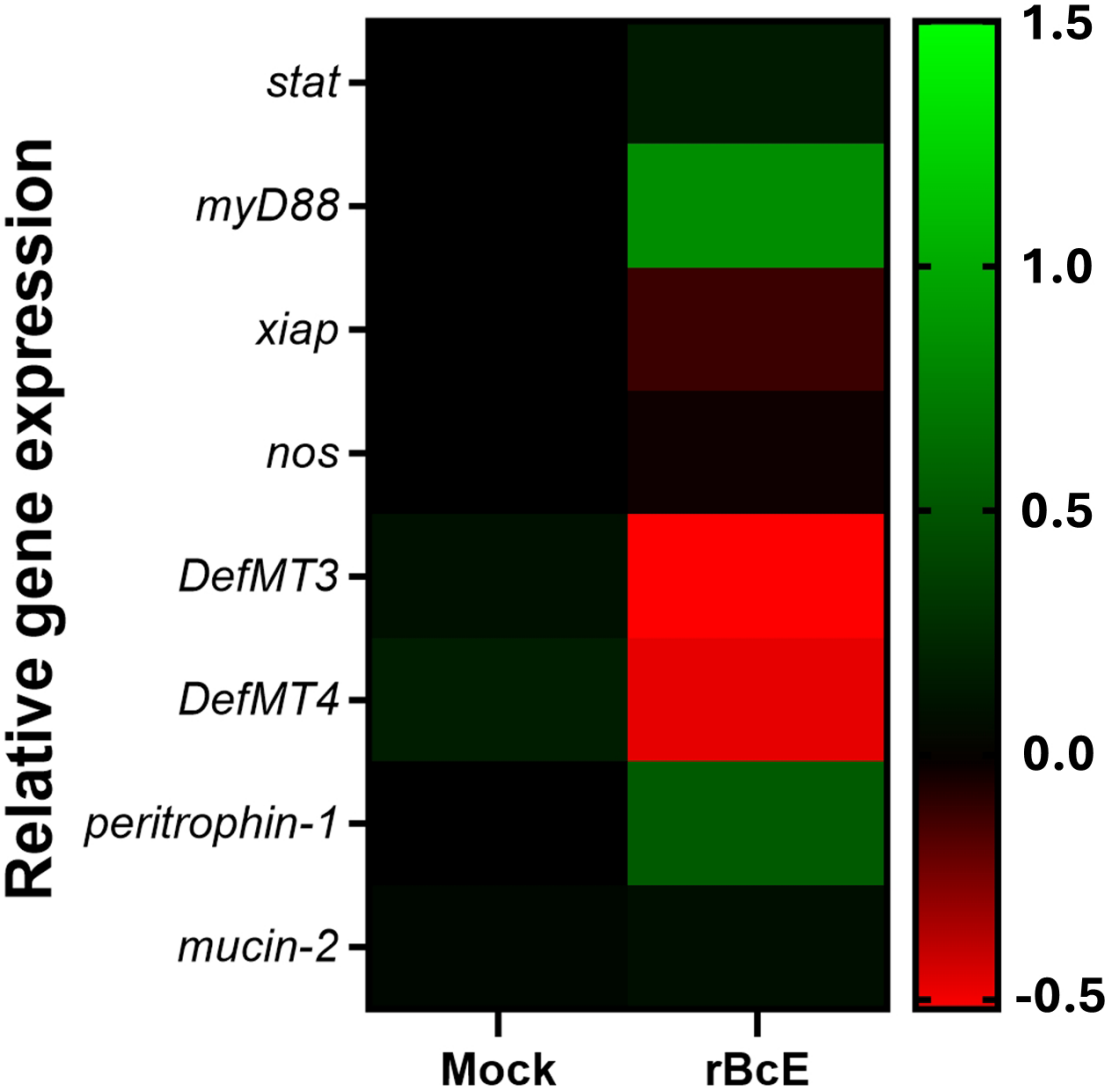
Heat map showing alterations in the expression profiles of the representative immune and structural genes between ticks fed with heat-inactivated (mock) or active r*Bc*E protein (*p* > 0.05). Transcription was assessed by RT-qPCR at 48 hours post-feeding and the data were normalized to the tick *elf-1a* housekeeping gene. Data represent mean values of three biological replicates.

Because the disruption of the arthropod gut barrier may induce an immune response in the epithelial cells (Kuraishi et al., 2011; Yang et al., 2014; Narasimhan et al., 2017; Rodgers et al., 2017; Talyuli et al., 2023), we sought to examine if specific immune pathways or components are involved in observed *B. afzelii* reduction in ticks. Expression profiles of the selected putative genes representing key factors of the JAK/STAT (*stat*), Toll (*myD88*), and IMD (*xiap*) immune signaling pathways, free radical defense (*nos*), antimicrobial peptides (*DefMT3*, *DefMT4*), and structural components of the PM (*peritrophin-1*, *mucin-2*) (Smith and Pal, 2014) were assessed by RT-qPCR. While transcript levels of *stat*, *xiap*, *nos*, and *mucin-2* were not altered, *myD88* and *peritrophin-1* genes were upregulated in the guts 48 hours after oral administration of the purified r*Bc*E protein. Expression levels of two defensins (*DefMT3*, *DefMT4*) were decreased in treated ticks compared to the mock control (**Figure 5**). These results imply that the degradation of the PM by *Bc*Enhancin affects the expression profile of the gut immune genes.

### 3.5 *Bc*Enhancin treatment influences total bacterial load and composition in infected tick guts

**Figure 6:**
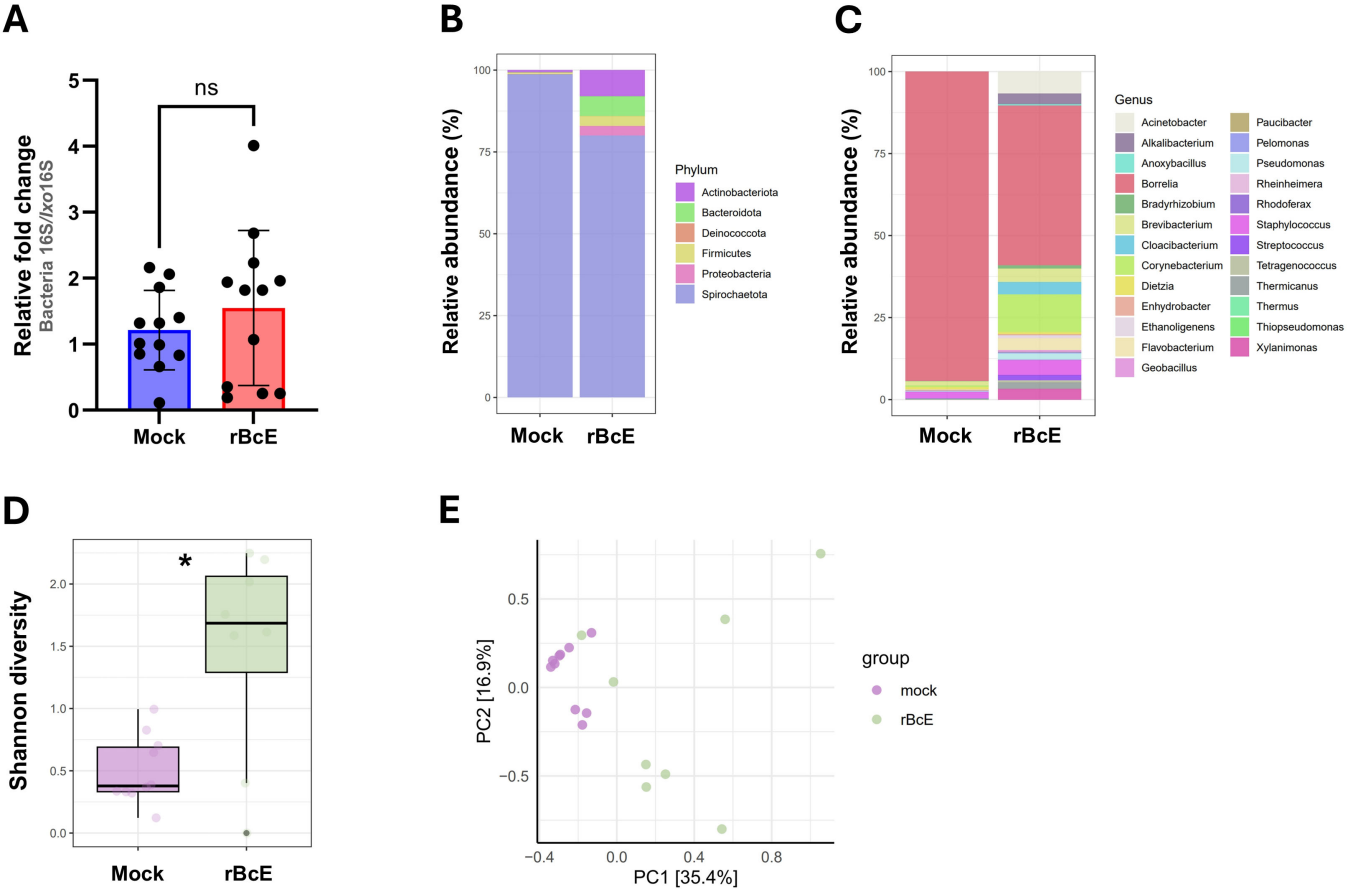
The tick PM is important for maintaining gut microbial homeostasis. **(A)** Total bacteria load in the guts of ticks fed with heat-inactivated (mock) or active r*Bc*E protein assessed by conventional PCR using universal 16S rRNA primers. Statistical significance was evaluated by a non-parametric Mann-Whitney *U* test (ns, not significant). **(B, C)** Taxonomic composition and relative abundance of bacterial communities in control and treated ticks at the taxonomic rank of phylum and genus. Only genera with relative abundance > 1% were considered. **(D)** Alpha diversity measured by Shannon diversity index (Student’s *t*-test, ^*^ *p* ≤ 0.05). **(E)** Principal component analysis (PCA) based on Bray-Curtis distances of bacterial communities between the groups, displayed by different colored dots.

As *Bc*Enhancin affected the gut protective barrier and immunity, we speculated that these changes might influence the microbiome composition. To address this, we first quantified the total bacterial burden by 16S rRNA gene-targeted qPCR. Ticks fed with heat-inactivated r*Bc*E had lower bacterial load 48 h after feeding compared to those fed with the intact protein, but the difference was not statistically significant (Mann Whitney *U* test, *p* = 0.5043, **Figure 6A**). Samples were then subjected to 16S rRNA gene amplicon sequencing to investigate the differences in bacterial diversity and composition between the groups. Six samples, two from the control group and four from the r*Bc*E group were excluded from the study due to inadequate reads. Amplicon sequence variants (ASVs) attributed to *Candidatus* Midichloria sp. were also removed from the data set because this endosymbiont primarily resides in ovaries (Olivieri et al., 2019) and we would not expect it to be part of the active gut microbiome. Gut bacterial community composition differed between the groups at both the phylum and the genus level (**Figures 6B, C**; **Supplementary Figures S5A, B**). The differences in composition were mainly due to alterations in the relative abundances of *Borrelia*, *Acinetobacter*, *Brevibacterium*, *Flavobacterium*, *Staphylococcus*, *Pseudomonas*, and *Corynebacterium* (**Figures 6C**; **Supplementary Figure S5B**). Alpha diversity was significantly different between the groups, with ticks from the r*Bc*E group having a higher Shannon diversity compared to control ticks (Student’s *t*-test, adjusted *p* = 0.0127, **Figure 6D**). Multivariate analysis of variance revealed significant differences in microbiome composition (PerMANOVA, *F* = 6.6083, adjusted *p* = 0.0001) and Principal component analysis (PCA) of microbial relative abundances indicated separation of clusters (**Figure 6E**). However, the difference in alpha (Student’s *t*-test, adjusted *p* = 0.854) and beta (PerMANOVA, *F* = 0.9818, adjusted *p* = 0.463) diversities between the groups was not significant after *Borrelia* ASVs were removed from the dataset (**Supplementary Figure S6**), suggesting that the alterations in microbiome composition was driven largely by *B. afzelii* abundance.

## 4 Discussion

Successful pathogen infection and persistence within a tick vector depends on the complex interactions between the host, its microbiome, and the pathogen (Kurokawa et al., 2020). The gut environment is profoundly influenced by residential microbes, which can either promote or antagonize pathogen colonization and its subsequent transmission to the vertebrate host (Kurokawa et al., 2020; Narasimhan et al., 2021; Wu-Chuang et al., 2021). Therefore, understanding the functional consequences of the tripartite interactions and the mechanisms by which certain microbial components of the gut microbiome might affect *Borrelia* infection success is of great research interest and could spur novel strategies to control both LB and its tick vector.

In the present study, we showed that *Bc*Enhancin derived from a tick-associated *Bacillus* bacterium limits *B. afzelii* persistence in *I. ricinus* females by interfering with the structural organization of the PM, further supporting the critical role of this physical barrier in the protection of spirochetes. The intact PM might be particularly important for *B. afzelii* infection success considering that this species, unlike the North American *B. burgdorferi* strains vectored by *I. scapularis*, remains in the tick gut until it is transmitted to the vertebrate host by regurgitation of spirochetes from the gut lumen (Pospisilova et al., 2019). Its integrity is largely influenced by the gut microbiome, which regulates the expression of peritrophins by the activation of the JAK-STAT signaling pathway (Narasimhan et al., 2014). Our data, however, suggest that *Bc*Enhancin induces structural changes in the PM by degrading the protective mucopolysaccharide layer, as seen by PAS staining of the tick gut sections, but this needs to be further confirmed by *in vitro* digestion assay with recombinant *I. ricinus* mucins. The similar mucin-degrading effect and destruction of the PM have been reported in other arthropods, including mosquitoes exposed to enhancins encoded by *B. cereus*, *Bacillus thuringiensis*, and *Serratia marcescens* (Fang et al., 2009; Wu et al., 2019). These bacterial enhancins also demonstrate the ability to digest intestinal mucins *in vitro* by cleaving the *O*-glycosylated sites (Fang et al., 2009; Wu et al., 2019). Mucins are heavily *O*-glycosylated proteins that, along with peritrophins, line the invertebrate PM and maintain its structural and functional integrity (Wu et al., 2019; Kozelková et al., 2023). Proteomic and transcriptomic profiling of *I. ricinus* guts revealed several putative mucins and peritrophins, some of which were upregulated upon infection with *B. afzelii* (Mahmood et al., 2021; Kozelková et al., 2023). Moreover, borrelial pathogens exploit chitobiose from the tick PM as an energy source and a building block for the bacterial cell wall (DeHart et al., 2021). These results may indicate that *Borrelia* induces the formation of the PM to enhance its colonization and persistence within the tick vector. By contrast, the compromised structure of the PM appears to promote infection with some intracellular tick-borne pathogens, such as *Anaplasma phagocytophilum*, *Theileria* sp., and *Babesia microti* by facilitating the pathogen migration from the gut to salivary glands (Abraham et al., 2017; Jalovecka et al., 2018; Wei et al., 2021). Consistent with these observations, higher abundances of *Bacillus* species in *Anaplasma*-positive *I. ricinus* (Lejal et al., 2021) and *Theileria*-positive *Rhipicephalus microplus* (Adegoke et al., 2020) ticks may suggest that members of this bacterial genus enhance the tick susceptibility to infections, possibly by interacting with the PM.

We also observed changes in the expression profiles of gut immune response genes in ticks with the compromised PM, which might affect *B. afzelii* colonization. The highest transcription level among selected genes was recorded for a gene encoding myD88 adapter protein. MyD88 is a key component of the Toll signaling pathway, which is predominantly induced by Gram-positive bacteria and fungi in *Drosophila* flies (Michel et al., 2001; Rutschmann et al., 2002), but the importance of Toll activation in controlling bacterial infections in ticks is poorly understood. In this context, the increased expression of *myD88* positively correlated with the increased relative abundance of Gram-positive bacteria such as *Staphylococcus*, *Streptococcus*, *Corynebacterium*, and *Brevibacterium* in r*Bc*E-fed ticks. Narasimhan and colleagues (2017) report similar findings in *I. scapularis* ticks after the abrogation of a gene encoding the PIXR gut protein with a Reeler domain. Specifically, knockdown of *pixr* impaired the structural integrity of the PM, decreased *B. burgdorferi* loads, and resulted in the elevated expression of another transcription factor of the Toll pathway called *dorsal*. Several immune pathways or their components, including myD88 have been shown to be involved in controlling the initial skin colonization by *Borrelia* spirochetes (Bockenstedt et al., 2006), suggesting that the immune response mediated by myD88 or some of the downstream Toll effector molecules might play a role in the clearance of *B. afzelii* from the guts of I*. ricinus*. Interestingly, ticks exposed to r*Bc*E showed decreased expression of two selected defensins that are typically expressed by *I. ricinus* gut cells (Tonk et al., 2014). Metalloproteases produced by certain bacterial and protozoan pathogens are known for their ability to inactivate multiple antimicrobial peptides, including defensins (Belas et al., 2004; Kulkarni et al., 2006). Consequently, it is possible that *Bc*Enhancin suppresses the activity of tick defensins to prevent bacterial cell death.

The breakdown in the integrity of the PM was also associated with a reduced load of *Borrelia*, but the abundances of other taxa were not significantly affected. This result suggests that the PM plays a selective role in limiting *Borrelia* burden in the tick gut, while having little to no effect on bacterial commensals that inhabit the intraperitrophic (luminal) space. According to our observations and those of previous studies (Kariu et al., 2013; Narasimhan et al., 2014; Yang et al., 2014, Yang et al., 2021), we hypothesize that the growth of *Borrelia* spirochetes is inhibited when they are exposed to luminal bacteria and/or their toxins in ticks with an impaired gut barrier.

In summary, our study uncovered the influence of residential gut microbes on tick-spirochete interaction dynamics and confirmed the importance of the PM for *B. afzelii* infection. While this knowledge moves the field beyond a descriptive understanding of the tick gut microbiome, further studies are required to establish a correlative relationship between enhancin-containing bacteria and resistance to *Borrelia* colonization of the tick gut under natural conditions. Our results suggest that targeting structural components of the tick gut holds potential for the development of alternative tools to control LB. Genetic modification of symbiotic or commensal gut-colonizing microbes to express effector molecules that can inhibit pathogen development within the vector has been recently proposed as an efficient strategy for the control of tick-borne diseases (Mazuecos et al., 2023). Therefore, using PM-degrading proteins in paratransgenesis represents a promising option that should be investigated in future studies.

## Data Availability

The datasets presented in this study can be found in online repositories. The names of the repository/repositories and accession number(s) can be found at: https://www.ncbi.nlm.nih.gov/, PRJNA1086846. Nucleotide sequences of the 16S rRNA gene from bacterial isolates have been deposited in GenBank^®^ with accession numbers PP446093-PP446122. The *B. cereus* LTG-1 enhancing gene sequence is available under accession number PP444694.
