# Supplementary material for: A metalloprotease secreted by an environmentally acquired gut bacterium hinders Borrelia afzelii colonization in Ixodes ricinus
Source: Front Cell Infect Microbiol. 2024 Oct 10;14:1476266. doi: 10.3389/fcimb.2024.1476266 (PMC11499241; doi:10.3389/fcimb.2024.1476266)
Supplement: Supplementary file 1 [file DataSheet1.docx]

***Supplementary information***


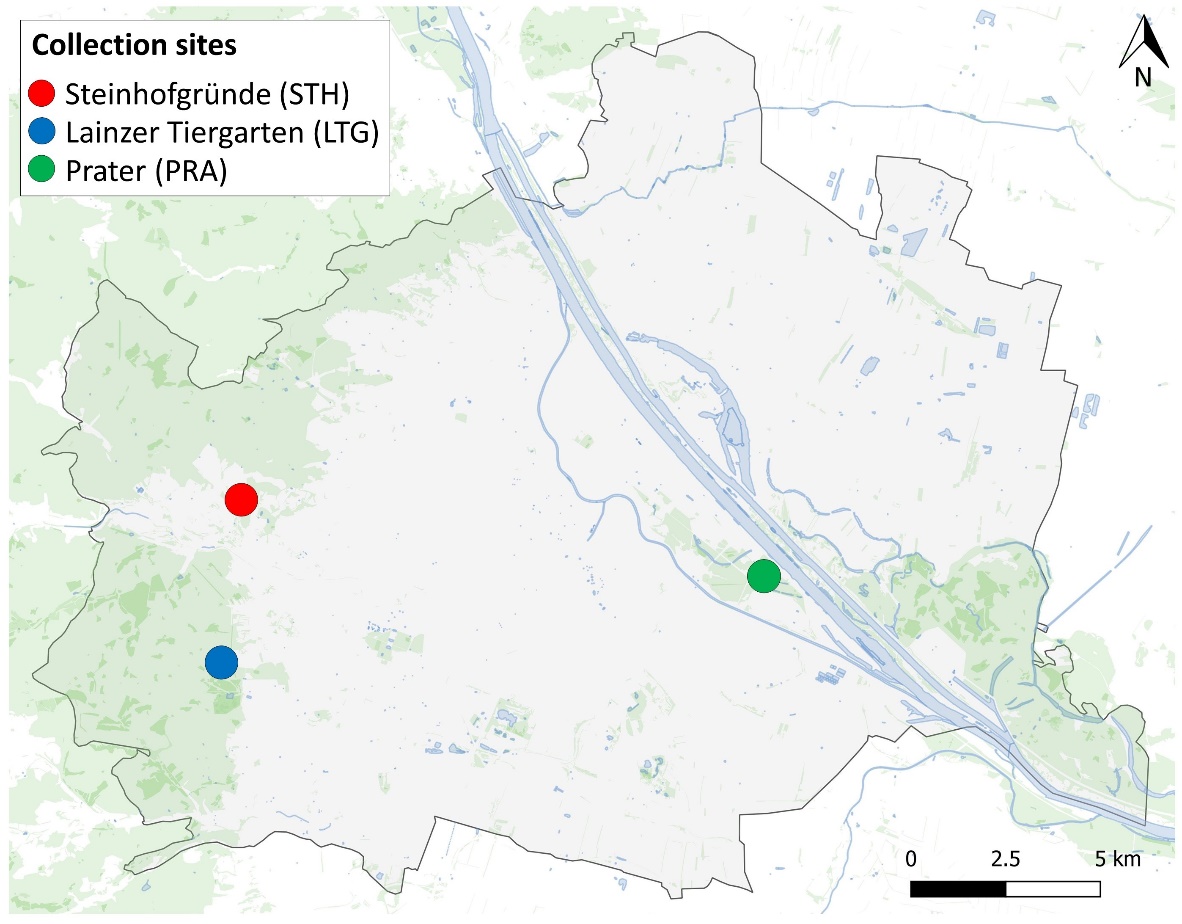


**Figure S1**. Location of the sampling sites in the city of Vienna, Austria. Coordinates: STH - Lat: 48°12´32.796´´ (N), Lon: 16°16´21.468´´ (E), LTG - Lat: 48°10´20.712´´ (N), Lon: 16°15´4.248´´ (E), PRA - Lat: 48°11´40.344´´ (N), Lon: 16°26´0.384´´ (E). Figure was created with [QGIS 3.4](https://www.qgis.org/).


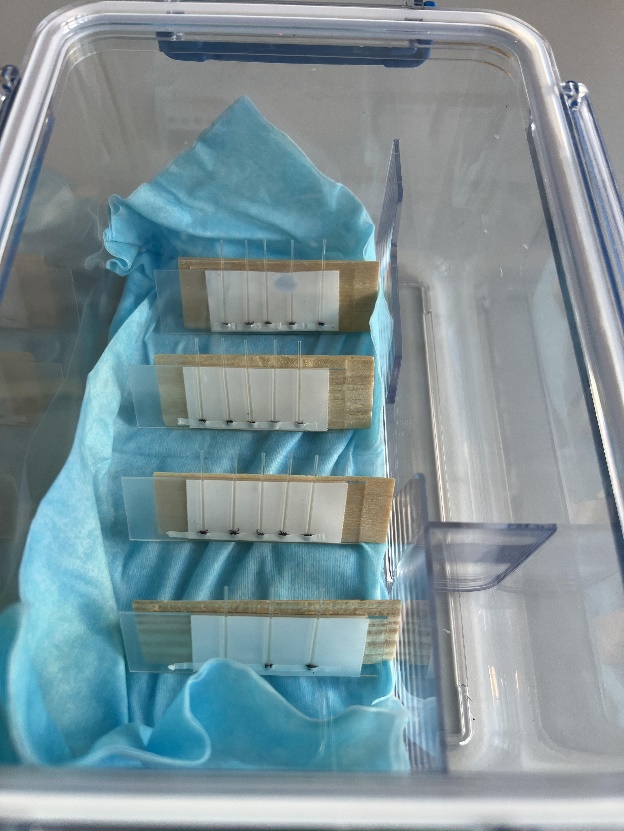


**Figure S2.** Tick infection with *Borrelia afzelii* RS 163_11i via capillary feeding.


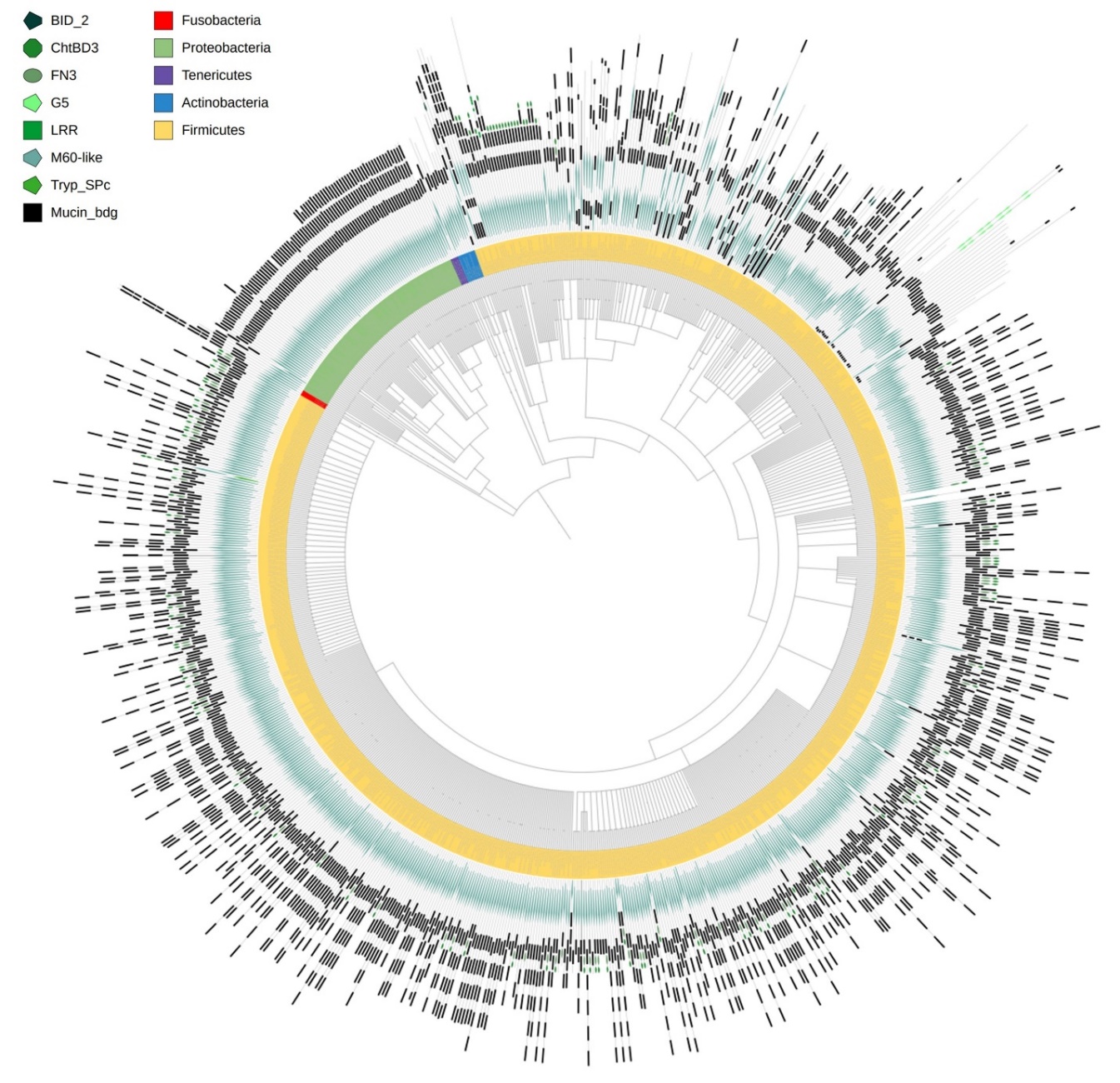


**Figure S3.** Protein domain architecture and distribution of enhancins across Bacteria superkingdom. The tree is generated based on the NCBI taxonomy in iTOL. The analysis revealed 900 proteins that are highly conserved across evolutionarily-distant bacterial taxa that colonize mammalian mucosal surfaces and invertebrate digestive tract. Apart from the M60-like (PF13402) and Mucin_bdg (PF03272) domains, which are the defining traits of all known mucin-degrading enhancins, some proteins from this group contain additional domains likely involved in cell adhesion (BID_2, FN3), binding and degradation of chitin (ChtBD3), N-acetylglucosamine binding (G5), proteolysis (Tryp_SPc), and protein-protein interactions (LRR).


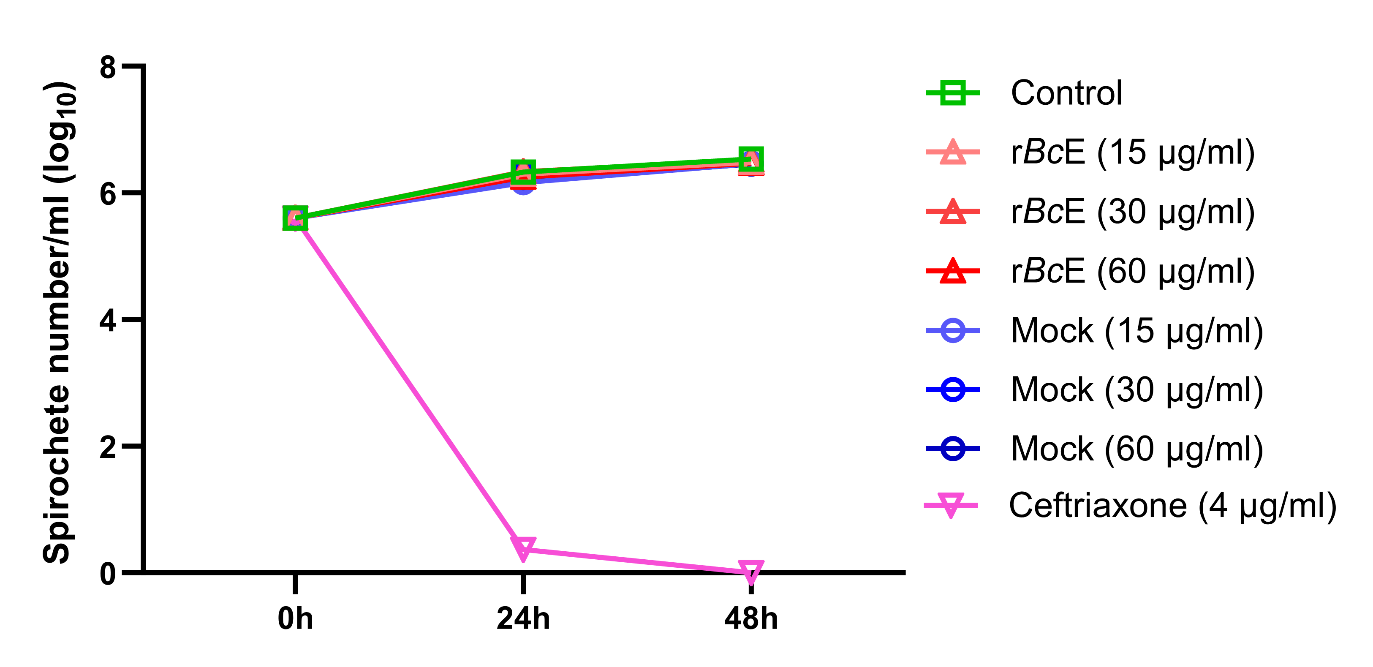
**Figure S4.** Bactericidal assay. Growth of *B. afzelii* RS 163_11i was assessed after 24 and 48 hours of incubation with ceftriaxone (positive control) or different concentrations of either active or heat-inactivated r*Bc*E protein. Untreated bacterial culture served as a negative control. Data represent mean values of three technical replicates. Exposure to r*Bc*E did not affect the viability and morphology of the spirochetes *in vitro*.


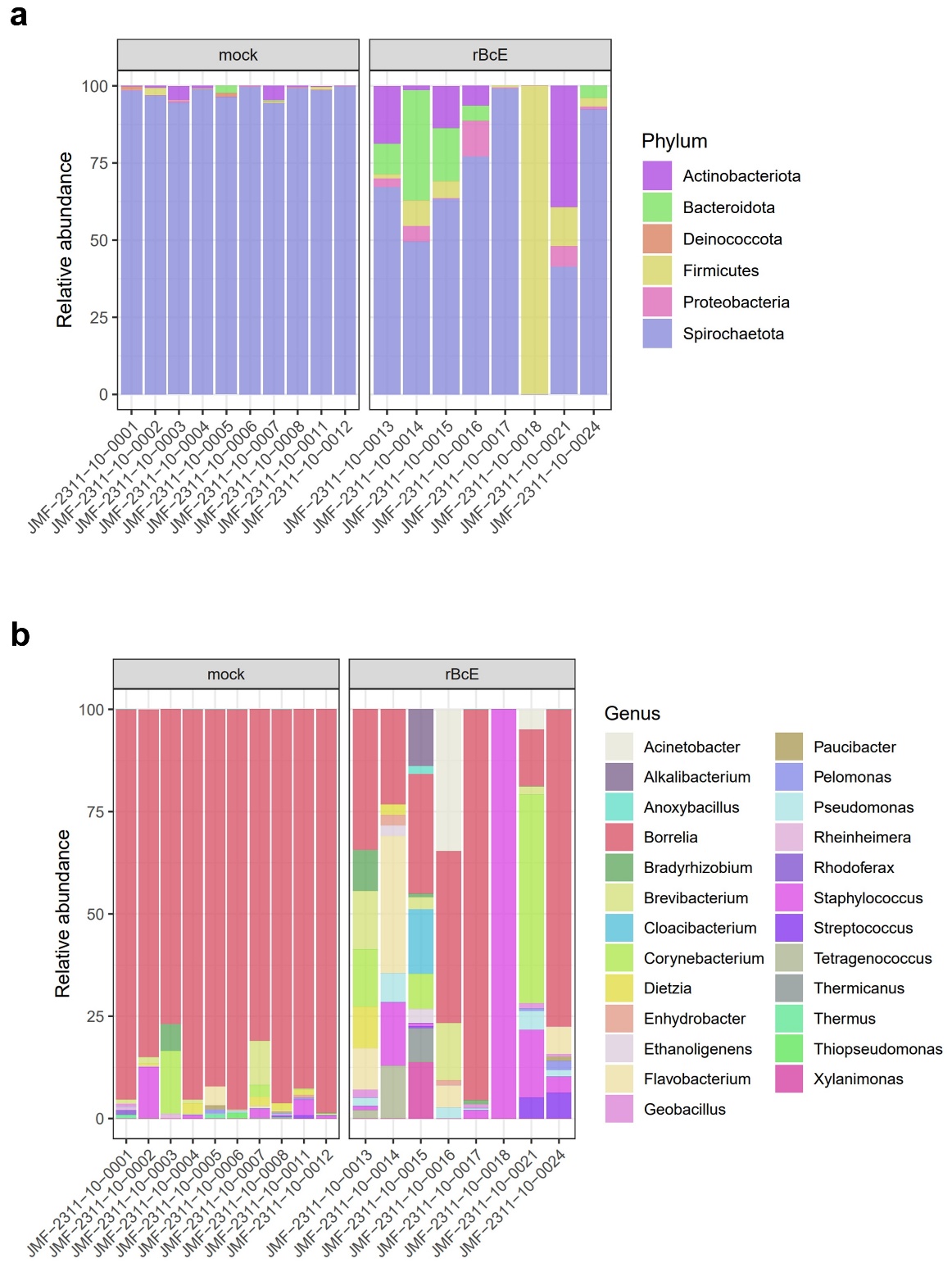


**Figure S5.** The relative abundance of gut bacteria at the phylum level (**A**) and the genus level (**B**) in individual tick samples. Only genera with relative abundance > 1% were considered.


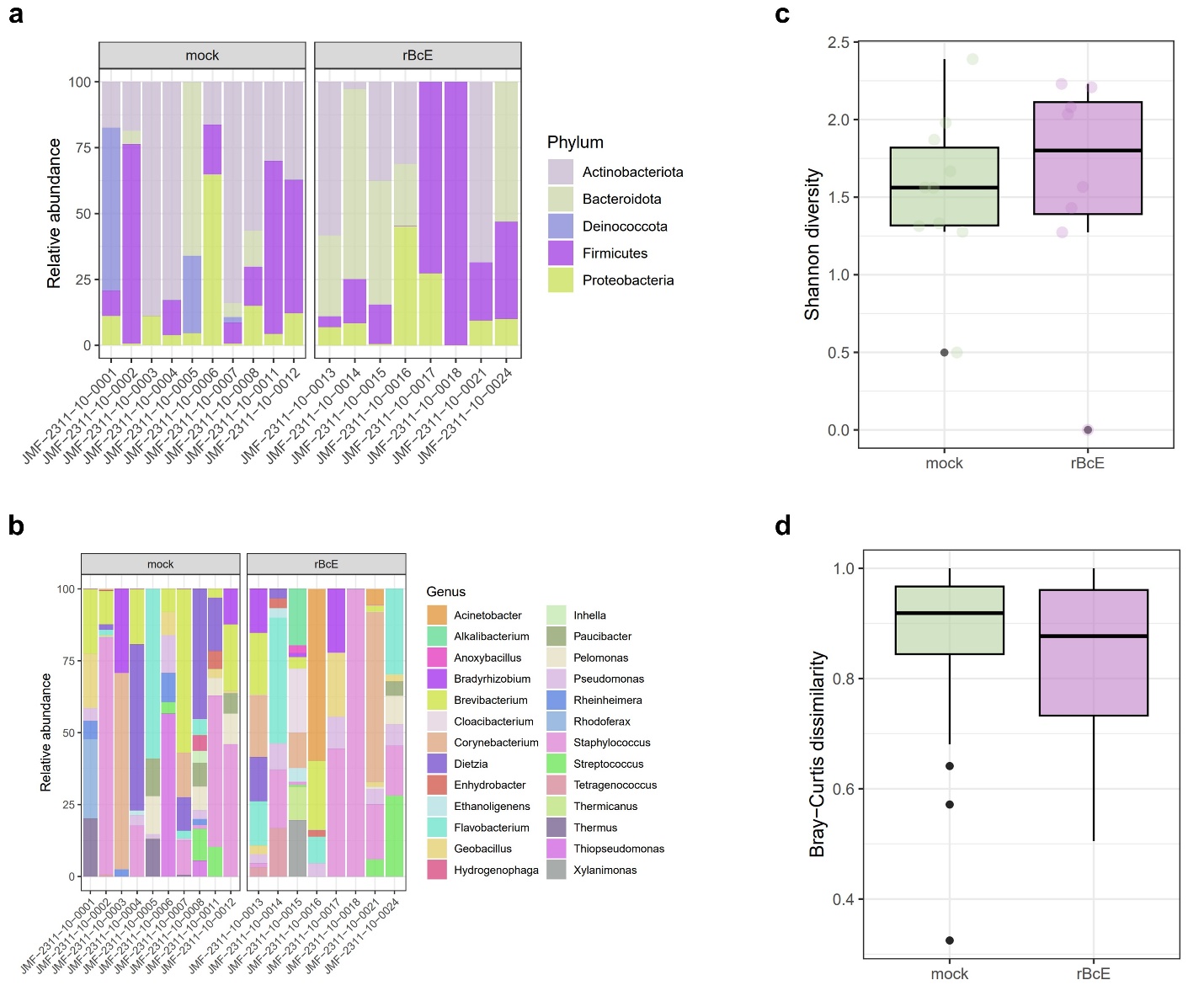
**Figure S6.** The relative abundance of gut bacteria at the phylum level (**A**) and the genus level (**B**) in individual tick samples after removal of *Borrelia* ASVs. **(C, D)** Alpha and beta diversity measured by Shannon diversity and Bray-Curtis dissimilarity index, respectively.

**Table S1.** Primers and probes used in the present study.

| **Target** | **Name** | **Sequence (5´ - 3´)** | **Tm** | **bp** | **Reference** |
| --- | --- | --- | --- | --- | --- |
| **Probe-based qPCR** | | | | | |
| *Ixodes* 16S | Ixo16S-F  Ixo16S-R  Ixo16S-Probe | AAAAAAATACTCTAGGGATAACAGCGTAA  ACCAAAAAAGAATCCTAATCCAACA  HEX- TTTTGGATAGTTCATATAGATAAAATAGTTTGCGACCTCG -BHQ1 | 60 | 98 | Becker et al., 2023 |
| *Borrelia* flagellin | FlaF1A  FlaR1  Fla Probe1 | AGCAAATTTAGGTGCTTTCCAA  GCAATCATTGCCATTGCAGA  FAM- TGCTACAACCTCATCTGTCATTGTAGCATCTTTTATTTG -BHQ1 | 60 | 174 | Schwaiger et al., 2001 |
| *Bacillus cereus* 16S | Bc16S-F  Bc16S-R  Bc16S-Probe | GCGGCGTGCCTAATACATGC  CTCAGGTCGGCTACGCATCG  FAM- TCGAGCGAATGGATTAAGAGCTTGC -BHQ1 | 60 | 267 | Reekmans et al., 2009 |
| **qPCR** | | | | | |
| Bacteria 16S | 341f  785r | CCTACGGGNGGCWGCAG  GACTACHVGGGTATCTAATCC | 55 | 444 | Klindworth et al., 2013 |
| **Standard PCR + sequencing** | | | | | |
| Bacteria 16S | 27f  1492r | AGAGTTTGATCMTGGCTCAG  GGTTACCTTGTTACGACTT | 51 | 1463 | Galkiewicz and Kellogg, 2008 |
| *enhancin* | BcEnhc-F  BcEnhc-R | TTGGATATTTCAGGCGGGCA  TGCAAAAGCTTCATCGCCAG | 60 | 952 | This study |
| **RT-qPCR** | | | | | |
| *enhancin* | EnhcRT-F  EnhcRT-R | ACTTAGAGCAAAAGCGGGCT  TGTCCCTTCCATTTCCTTCGT | 60 | 137 | This study |
| *elongation factor*  (E3SS18) | Irelf1a_f  Irelf1a_r | CCAAGACCTTTGTTGCTGGC  CAGGCTGGTGTCTGCGATAT | 63 | 91 | This study |
| *peritrophin-1*  (V5H8Q8) | per1_F  per1_R | CACCGAGCCGTCTTGTCC  ACTTTCCGGTGACCTTGTTGA | 63 | 152 | This study |
| *mucin-2-like*  (A0A147BFP1) | muc2-F  muc2-R | CAGAGGCCATCCGAGGAATC  TCGGATAGGCCTCCTCGTAG | 63 | 106 | This study |
| *stat*  (V5H6X7) | Irstat-F  Irstat-R | GAGTATCACCAGGCCACGAG  CCACCGCCAACCTTGAATTG | 63 | 149 | This study |
| *myD88*  (V5GVQ3) | myD88-F  myD88-R | TCTGAAGTCCCACGAATGCC  CATCCTGATCATGCCGTCGA | 63 | 124 | This study |
| *xiap*  (V5H7Q7) | xiap-F  xiap-R | TGTCACAAGAACGATCGCCA  AAACTTGGAGAGTCGCGGAG | 63 | 85 | This study |
| *nos*  (A0A147BLV2) | nos-F  nos-R | GAAGTGGAGCGTCAGGTTCA  AACACCTGCAGTTTGGACCA | 63 | 134 | This study |
| *DefMT3*  (A0A089VRA3) | Irdef3-F  Irdef3-R | TTTTTATTGACAGGCCGCGG  GCTTTTCTCCCGAAGCTCCT | 63 | 86 | This study |
| *DefMT4*  (A0A089VKM9) | Irdef4-F  Irdef4-R | CGCCGACTTTTCAAACGACA  ATGGGCAGTAGTAACCACCG | 63 | 95 | This study |
| **FISH** | | | | | |
| *Borrelia* spp. | Borr4_DOPEcy3 | Cy3- CCAACACCTCACAGCACGAGC -Cy3 | 20% FA | | Hammer et al., 2001 |

**Table S2.** List of cultivable bacterial isolates, their growth conditions, and origin.

| **Species** | **Gram stain** | **Phylum** | **Family** | **Condition (°C)** | **Isolation source** | **Main source** |
| --- | --- | --- | --- | --- | --- | --- |
| *Bacillus cereus* | Gram (+) | Firmicutes | Bacillaceae | Aerobic (37) | N, F | Soil |
| *Bacillus licheniformis* | Gram (+) | Firmicutes | Bacillaceae | Aerobic/anaerobic (22, 37) | N, F | Soil |
| *Metabacillus idriensis* | Gram (+) | Firmicutes | Bacillaceae | Aerobic (37) | F | Blood, soil |
| *Metabacillus halosaccharovorans* | Gram (+) | Firmicutes | Bacillaceae | Aerobic (37) | F | Soil, water |
| *Peribacillus frigoritolerans* | Gram (+) | Firmicutes | Bacillaceae | Aerobic (22, 37) | N, F | Soil |
| *Micrococcus luteus* | Gram (+) | Actinobacteria | Micrococcaceae | Aerobic (22, 37) | N, F | Skin, soil, water |
| *Kocuria indica* | Gram (+) | Actinobacteria | Micrococcaceae | Aerobic (37) | F | Soil |
| *Staphylococcus epidermidis* | Gram (+) | Firmicutes | Staphylococcaceae | Aerobic (37) | N, F | Skin |
| *Staphylococcus caprae*/*S. capitis* | Gram (+) | Firmicutes | Staphylococcaceae | Anaerobic (22, 37) | F | Skin |
| *Bacillus mycoides* | Gram (+) | Firmicutes | Bacillaceae | Aerobic/anaerobic (22, 37) | N | Soil |
| *Peribacillus butanolivorans* | Gram (+) | Firmicutes | Bacillaceae | Aerobic (22) | N | Soil |
| *Bacillus proteolyticus* | Gram (+) | Firmicutes | Bacillaceae | Anaerobic (37) | N | Soil |
| *Lysinibacillus* sp. | Gram (+) | Firmicutes | Planococcaceae | Aerobic (22) | N | Unknown |
| *Bacillus* sp. 1 | Gram (+) | Firmicutes | Bacillaceae | Aerobic (37) | N | Unknown |
| *Bacillus* sp. 2 | Gram (+) | Firmicutes | Bacillaceae | Aerobic (37) | N | Unknown |
| *Priestia aryabhattai* | Gram (+) | Firmicutes | Bacillaceae | Aerobic (22) | N | Soil |
| *Paenibacillus profundus* | Gram (+) | Firmicutes | Paenibacillaceae | Aerobic (37) | N | Soil |
| *Neobacillus niacini* | Gram (+) | Firmicutes | Bacillaceae | Aerobic (37) | N | Soil |
| *Peribacillus simplex* | Gram (+) | Firmicutes | Bacillaceae | Aerobic (37) | N, M | Soil |
| *Lysinibacillus xylanilyticus* | Gram (+) | Firmicutes | Planococcaceae | Aerobic (37) | N | Soil |
| *Curtobacterium flaccumfaciens* | Gram (+) | Actinobacteria | Microbacteriaceae | Aerobic (22) | N, F, M | Plant debris |
| *Paenibacillus* sp. | Gram (+) | Firmicutes | Paenibacillaceae | Aerobic (22) | N, M | Unknown |
| *Solibacillus cecembensis* | Gram (+) | Firmicutes | Planococcaceae | Aerobic (22) | N | Soil |
| *Staphylococcus hominis* subsp. *novobiosepticus* | Gram (+) | Firmicutes | Staphylococcaceae | Anaerobic (37) | N | Skin, blood |
| *Metasolibacillus* sp. | Gram (+) | Firmicutes | Planococcaceae | Aerobic (22) | N | Unknown |
| *Staphylococcus warneri* | Gram (+) | Firmicutes | Staphylococcaceae | Aerobic (37) | M | Skin |
| *Paenibacillus xylanivorans* | Gram (+) | Firmicutes | Paenibacillaceae | Aerobic (37) | N | Soil |
| *Bacillus toyonensis* | Gram (+) | Firmicutes | Bacillaceae | Anaerobic (22) | N | Soil |
| *Curtobacterium citreum* | Gram (+) | Actinobacteria | Microbacteriaceae | Aerobic (22) | N | Soil |
| *Microbacterium saperdae* | Gram (+) | Actinobacteria | Microbacteriaceae | Aerobic (22) | N | Arthropod |

N - nymphs, F - females, M - males

**References**

1. Becker ,N.S., Rollins, R.E., Stephens, R., Sato, K., Brachmann, A., Nakao, M., et al. (2023). *Candidatus* Lariskella arthopodarum endosymbiont is the main factor differentiating the microbiome communities of female and male *Borrelia*-positive *Ixodes persulcatus* ticks. *Ticks Tick Borne Dis*. 14, 102183. doi: 10.1016/j.ttbdis.2023.102183
2. Schwaiger, M., Péter, O., Cassinotti, P. (2001). Routine diagnosis of *Borrelia burgdorferi* (sensu lato) infections using a real-time PCR assay. *Clin. Microbiol. Infect*. 7, 461-9. doi: 10.1046/j.1198-743x.2001.00282.x
3. Reekmans, R., Stevens, P., Vervust, T., De Vos, P. (2009). An alternative real-time PCR method to detect the *Bacillus cereus* group in naturally contaminated food gelatine: a comparison study. *Lett. Appl. Microbiol*. 48, 97-104. doi: 10.1111/j.1472-765X.2008.02495.x
4. Klindworth, A., Pruesse, E., Schweer, T., Peplies, J., Quast, C., Horn, M., *et al*. (2013). Evaluation of general 16S ribosomal RNA gene PCR primers for classical and next-generation sequencing-based diversity studies. *Nucleic Acids Res*. 41, e1. doi: 10.1093/nar/gks808
5. Galkiewicz, J.P., Kellogg, C.A. (2008). Cross-kingdom amplification using bacteria-specific primers: complications for studies of coral microbial ecology. *Appl. Environ. Microbiol*. 74, 7828-31. doi: 10.1128/AEM.01303-08
6. Hammer, B., Moter, A., Kahl, O., Alberti, G., Göbel, U.B. (2001). Visualization of *Borrelia burgdorferi* sensu lato by fluorescence *in situ* hybridization (FISH) on whole-body sections of *Ixodes ricinus* ticks and gerbil skin biopsies. *Microbiology* (Reading) 147, 1425-1436. doi: 10.1099/00221287-147-6-1425
